# Extracellular electron uptake from carbon-based π electron surface-donors: oxidation of graphite sheets by *Sulfobacillus thermosulfidooxidans* probed by Raman and FTIR spectroscopies

**DOI:** 10.1039/c9ra03628h

**Published:** 2019-06-17

**Authors:** Charalampos Tselios, Marios Papageorgiou, Constantinos Varotsis

**Affiliations:** Cyprus University of Technology, Department of Environmental Science and Technology Lemesos Cyprus c.varotsis@cut.ac.cy +357 25002802

## Abstract

In this work we report Raman and FTIR evidence for extracellular electron uptake by *Sulfobacillus thermosulfidooxidans* from the solid phase carbon-based π-electron donor surface of graphite sheets. The primary step in the reaction is the intercalation of water on the surface of graphite followed by the formation of EPS and proceeds to form graphite oxide (GO) with a Raman *I*_D_/*I*_G_ = 0.3 ratio which represents the highest defect content in the carbon lattice reported by bio-oxidation process. We propose and discuss a direct extracellular electron transfer mechanism *via* outer membrane redox proteins for the electron transfer.

## Abstract 2

In this work we report Raman and FTIR evidence for extracellular electron uptake by *Sulfobacillus thermosulfidooxidans* from the solid phase carbon-based π-electron donor surface of graphite sheets.

## Graphical abstract

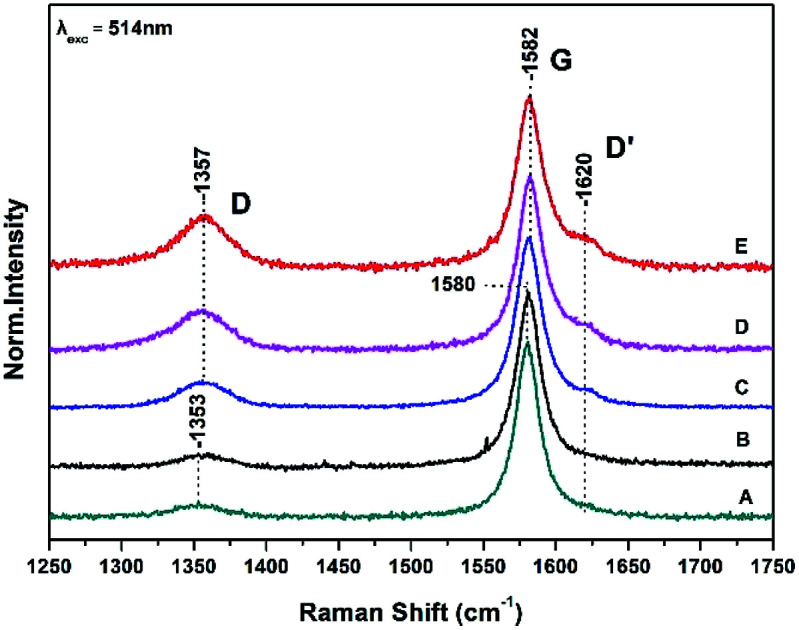

## 1. Introduction

Microbial fuel cells (MFCs) are devices that convert chemical energy into electricity *via* the extracellular transfer of electrons by selected bacteria.^1^ MFCs offer a sustainable alternative energy source that has multifunctional applications.^2^ Graphite and graphite fiber composite electrodes have been prepared as anodes in high substrate concentration MFCs and can be used to generate a higher voltage than traditional methods. The current output capability of MFCs is a function of temperature and the Microbial Extracellular Electron Transfer (MEET) mechanisms involved have been a focus for studies in microbial electrochemical technology (MET).^2,3^ In the field of bio-electrochemical systems, graphite plates are used in the manufacture of proton exchange membrane fuel cells.^4^ These fuel cells are being developed for transport applications as well as for stationary and portable fuel cell applications.^3^ In addition, graphite submicron particles are used as a possible catalyst support for polymer electrolyte membrane fuel cells.^3,5^

Three proposed MEET mechanisms with many applications in biotechnology including bio-electrochemical systems and in the field of hydrometallurgy exist: (1) direct extracellular electron transfer (EET) *via* outer membrane redox proteins. (2) direct EET *via* solid conductive matrix and (3) indirect EET *via* an electron mediator compound (*M*_red_/*M*_ox_).^6–8^ There is consensus for the existence of all three mechanisms but there are not available studies, which mechanism is the dominant one. The attachment of bacteria to the graphite surface is a necessary condition for the direct mechanism but not a satisfactory proof. The microorganisms in biofilms live in matrix of hydrated extracellular polymeric substances (EPS) that form their immediate environment which consist of polysaccharides, proteins, nucleic acids and lipids that provide the mechanical stability of biofilms and mediate their adhesion to surfaces.^7,8^*Thiobacillus ferrooxidans* is involved in electricity generation in microbial fuel cell systems acting as the cathode and by an unknown mechanism, oxidize graphite powder.^9^ It was proposed that the oxidation of graphite occurred in the periplasm space by the copper protein rusticyanin acting as the electron acceptor.^9^ In terms of the electron transfer mechanisms, direct and indirect mechanisms concerning the action of *T. ferrooxidans* with sulfide minerals have been proposed in the field of hydrometallurgy.^7,8^ Members of the genus *Sulfobacillus* are Gram-positive bacteria, moderately thermophilic acidophiles, metal-tolerant that promote sulfide mineral dissolution and occur in acidic habitats, hydrothermal vents, and in industrial hydrometallurgy operations.^10^ Genome analysis of *Sulfobacillus thermosulfidooxidans* revealed some features not yet found in other acidophiles such as the Gram-negative *A. ferrooxidans.*^10^ The blue copper protein sulfocyanin, sharing sequence characteristics with the *A. ferrooxidans* blue copper protein rusticyanin, is an important component of the electron transport chain during iron oxidation by *S. thermosulfidooxidans* and is involved in the electron transfer pathways during the bioleaching of Cu containing iron sulfides.^7,8,10^ In hydrometallurgy the role of the moderate thermophiles in complex Cu-ores bioleaching with the assistance of graphene was recently reported.^11^

The electron uptake mechanism from the solid phase electron donors to the microbes is critical to our understanding of the ecological and evolutionary implications of this process, as well as to the bio-electrosynthesis processes of graphite.^11^ The versatility of *S. thermosulfidooxidans* is an essential factor for the competitive success in the extreme acidic environments. Although most organisms use soluble oxidants and reductants, some microbes can access solid-phase materials as electron-acceptors or-donors *via* extracellular electron transfer.^6^ Many studies have focused on the reduction of solid-phase oxidants but little is known about electron uptake *via* microbial extracellular electron transfer, and almost nothing is known about the associated mechanisms. In the work described herein, we investigated the reaction of graphite sheets with *S. thermosulfidooxidans* under aerobic conditions by employing 514 nm micro-Raman excitation and FTIR microspectroscopy. We provide experimental evidence that graphite is oxidized and forms GO with *I*_D_/*I*_G_ = 0.3 which represents the highest defect content in the carbon lattice by a bio-oxidation process in much shorter times compared to those previously reported.^9^ The techniques illustrate the potential of the techniques to perform ultrasensitive chemical analysis on graphite sheets and biofilms, including the detection of different components and the determination of their relative abundance on the surface of graphite.^7,8,12,13^ The biofilm formation which mediates the adhesion of the microorganism to the surface of graphite sheets is demonstrated by the presence of FTIR bands in the 1100–1300 cm^−1^ bands and the formation of GO is characterized by the bands at 3347 and 3463 cm^−1^ which are attributed to the stretching vibration of –OH groups (V_O–H_) either from –OH groups of absorbed water or –OH groups formed during the oxidation. The introduction of oxygen containing groups between the sheets of graphite is the primary step towards the formation of GO which can be converted to graphene oxide and subsequently reduced to form reduced graphene oxide.

## 2. Experimental

### 2.1 Graphite bio-oxidation

Bio-interaction experiments were carried out under aseptic conditions using graphite sheets and a pure culture of *S. thermosulfidooxidans* (DSM 9293). Bacteria were inoculated into serum bottles containing 8 mL of growth medium provided by DSMZ and one graphite sheet (3 × 1 × 0.2 cm). Graphite sheets were purchased from GraphiteStore.com, IL. U.S.A. The graphite sheets were incubated with *S. thermosulfidooxidans* at 50 °C under shaking at 100 rpm for 3 days prior to the Raman and FTIR measurements. Control samples contained the same growth medium and graphite sheets without *S. thermosulfidooxidans*. The cells were alive for more than three days. Therefore, our measurements are from the interaction of live cells with the surface of the graphite sheets.

### 2.2 Characterization

Raman data were collected by a confocal LabRAM (HORIBA JobinYvon, Kyoto, Japan) equipped with a CCD detector and 1800 grooves per mm grating. The 514 nm excitation beam was provided by a Coherent Sapphire laser. Laser power was 20 mW and the total accumulation time for each measurement was 10 minutes. The FTIR spectra were recorded using a Tensor 27 infrared spectrometer coupled with the HYPERION 2000 microscope (Bruker). Measurements were collected in reflectance acquisition mode using a 15× IR objective and a gold mirror as a reference. Spectra acquisitions were obtained at a resolution of 4 cm^−1^ over the spectral range 800–4000 cm^−1^ using 32 scans. The sample compartment was purged with nitrogen gas. Raman and FT-IR experiments were performed immediately after the removal of the samples from the bottles containing the bacterial cultures and were not washed or dried after the bio-oxidation process.

## 3. Results and discussion

**Fig. 1:**
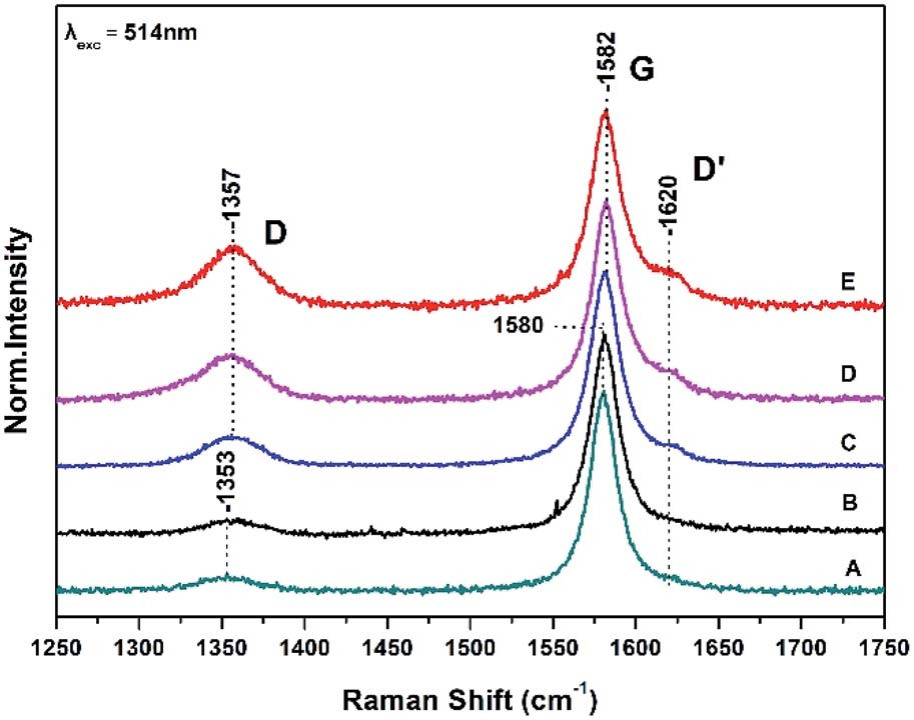
Raman spectra of graphite sheets (A), the surface of the bacteria growth medium–graphite sheets (B), and from three different spots (spectra C, D and E) from the surface of the *S. thermosulfidooxidans*–graphite sheets.

Fig. 1 shows Raman spectra of graphite sheets (A), the surface of the bacteria growth medium–graphite sheets (B), and from three different spots (spectra C, D and E) from the surface of the *S. thermosulfidooxidans*–graphite sheets.

The Raman spectra are characterized by the well characterized D and G bands.^14,15^ The D band at 1353 cm^−1^ is a breathing mode of A_1g_ symmetry involving phonons near the K zone boundary. The G mode at 1580 cm^−1^ originates from the in-plane vibration of sp^2^ carbon atoms and is double degenerate phonon mode (E_2u_ symmetry) at the Brillouin zone center. The frequencies, intensity ratio and FWHM of both the D and G Raman bands are in agreement with those previously reported.^9,14,15^

A ratio of the intensities of D and G bands (*I*_D_/*I*_G_) determines the relative defect content in the carbon lattice. The measured *I*_D_/*I*_G_ of pure graphite sheets is 0.07 in full agreement with previous measurements and remains constant in the control experiment which is consisted of the growth medium of the *S. thermosulfidooxidans*–graphite sheets (spectrum B) indicating that the bacteria growth material has no effect on the surface properties of graphite sheet.^9^ In spectra C, D, and E there is a 2–3 cm^−1^ frequency up-shift of both the D and G bands. The presence of the D′ at 1620 cm^−1^ in spectra C, D, and E indicates that there are some randomly distributed impurities or surface charges on the surface of graphite sheets and thus the G-peak splits into two peaks, the G-peak at 1582 cm^−1^ and the D′ peak at 1620 cm^−1^. The observed splitting is due to the interaction of localized vibrational modes of impurities with the extended phonon mode of graphite resulting in the observed splitting.^14,15^ It is a second-order process which is responsible for its dispersive nature and cause a strong dependence on the perturbation to the electronic structure and phonon structure of graphite.^14,15^ An increased ratio of *I*_D_/*I*_G_ is related to the formation of vacancies and defects in carbon lattice. The range of the (*I*_D_/*I*_G_) ratios of the treated graphite sheets we have observed in spectra C–D varies from 0.15 to 0.3. This means that the process is inhomogeneous and introduction of more oxygen-functional groups during the oxidation results in formation of more defects and vacancies in the final product-graphite oxide (GO). The full widths at half maximum (FWHM) of the G band which is 21 cm^−1^ is narrower that the chemically modified graphite powder (80 cm^−1^) indicating a lower level of disorder.^9^

**Fig. 2:**
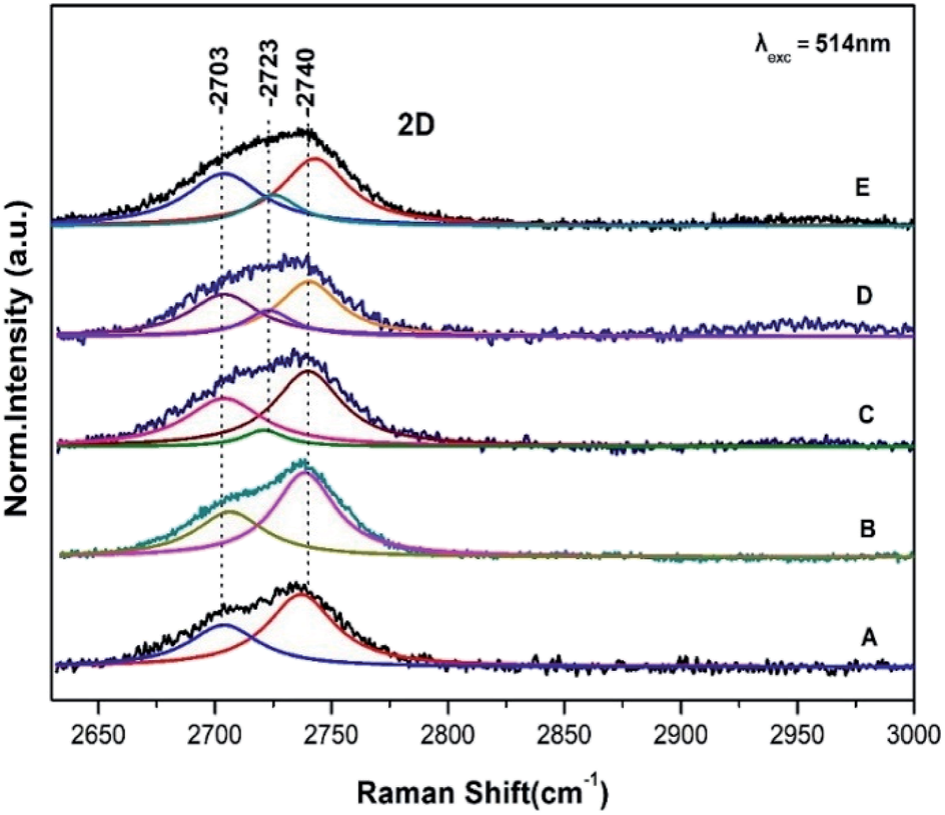
Raman spectra of graphite sheets (A), pristine control sample (B) and from three different spots (C, D and E) of the *S. thermosulfidooxidans*–graphite sheets. Same conditions as in Fig. 1.

Fig. 2 shows the corresponding Raman spectra in the 2600–3000 cm^−1^ range of those presented in Fig. 1 and are characterized of the 2D bands. The 2D band arises from a two phonon double resonance Raman process and is a key mode in identification of graphene layers.^14,15^ The 2D peak in graphite has two components 2D_1_ and 2D_2_ roughly 1/4 and 1/2 of *I*_G_ clearly demonstrated in spectra A and B by the peaks at 2703 and 2740 cm^−1^. The data in Fig. 2 (spectrum C) demonstrate that the intensities of the observed 2D_1_ at 2701 cm^−1^ and 2D_2_ at 2740 cm^−1^ are approximately 1/4 and 1/2 of the *I*_G_ presented in Fig. 1. Spectra D and E however demonstrate the presence of a new band at 2723 cm^−1^ which is developed on the expense of the 2740 cm^−1^ and thus the ratio of the 2D_1_ and 2D_2_ has been modified. The 2701 cm^−1^ band also indicates that the number of layers is much less than that of the graphite. In addition the *I*_D_/*I*_G_ ratio increases in order of spectra C, D and E. This is related to the formation of vacancies and defects in carbon lattice and indicates that the process is inhomogeneous and also that introduction of more oxygen-functional groups during the oxidation results in formation of more defects and vacancies in the final product. Three Lorentzian functions (2D_1_, 2D_2_, 2D_3_) are used for fitting the 2D-band of modified graphite surface using Levenberg–Marguardt algorithm.^16^ The fitting was performed after a linear baseline correction without limitations on any spectra parameter or further processing. Table 1 summarizes the fitting parameters, peak positions, FWHM of all bands observed in the Raman spectra of Fig. 1 and 2.

Frequencies of D, G, D′, 2D_1_, 2D_2_, 2D_3_, FWHM, *I*_D_/*I*_G_ and *I*_2D_2__/*I*_2D_1__

**Table:** 

	Position (cm^−1^)			FWHM (cm^−1^)			Ratio
	G	D	D′	G	D	D′	*I*_D_/*I*_G_
Graphite	1580	1353	1620	19	35	—	0.07
Control sample	1580	1354	1620	20	35	—	0.07
MG C	1582	1356	1620	21	36	—	0.15
MG D	1582	1356	1620	21	36	—	0.23
MG E	1582	1357	1620	22	35	—	0.30

**Table:** 

	Position (cm^−1^)			FWHM (cm^−1^)			Ratio
	2D_1_	2D_2_	2D_3_	2D_1_	2D_2_	2D_3_	*I*_2D_2__/*I*_2D_1__
Graphite	2703	2723	2737	29	—	34	—
Control sample	2703	2723	2739	30	—	32	—
MG C	2703	2723	2740	32	15	32	0.3
MG D	2703	2723	2740	34	15	31	0.6
MG E	2703	2724	2743	33	15	30	0.6

### 3.1 Disorder-induced D and D′

The presence of disorder in sp^2^ carbon is demonstrated by the increase intensity of the D band with the highest intensity observed in spectrum E. The frequency shift of the D band from 1353 cm^−1^ in graphite to 1357 cm^−1^ implies that the disorderness of graphite and can be attributed to the site-to-site variation in the number of next nearest neighbours, that is, when sp^2^ C atoms have sp^3^ C neighbors.^14,15^ The quantifying disorder is made by analyzing the *I*_D_/*I*_G_ intensity ratio between the disorder-induced D band and the Raman allowed G-band which varies from 0.15 to 0.3 (Table 1).

### 3.2 The G band

The G peak can split into two peaks, G and D′. The origin of the splitting is the localized vibrational modes of the *S. thermosulfidooxidans*–graphite complex interacts with the extended phonon modes of graphite resulting in the observed splitting. This means that introduction of more oxygen-functional groups during the oxidation results in more defects and vacancies in the final product. When the C–C bond lengths and angles are modified by strain, caused by the interaction with a substrate, the symmetry of graphite is affected.^14,15^ The FWHM of the G band increases with disorder, and our data indicate that this is the case in our samples.^14,15^

### 3.3 Dispersive 2D band

There is a strong coupling between the *I*_D_/*I*_G_, the D′ band and the formation of the new 2D band at 2723 cm^−1^. It should be noted that the component at 2703 cm^−1^ displays intensity fluctuations and in conjunction with the presence of the other 2D components it may attributed to the separation of the graphite sheets. The intensity *I*_D_/*I*_G_ ratio is directly related to the size of the sp^2^ phase organized in rings and gives an indirect indication of the sp^3^ content in the samples.^14,15^ The FWHM of the G band is sensitive to structural disorder *i.e.* an increase of the disorder is linked to higher sp^3^ content.^14,15^ Since there is a minor broadening of the G peak there is a slight disordering of the structure.

**Fig. 3:**
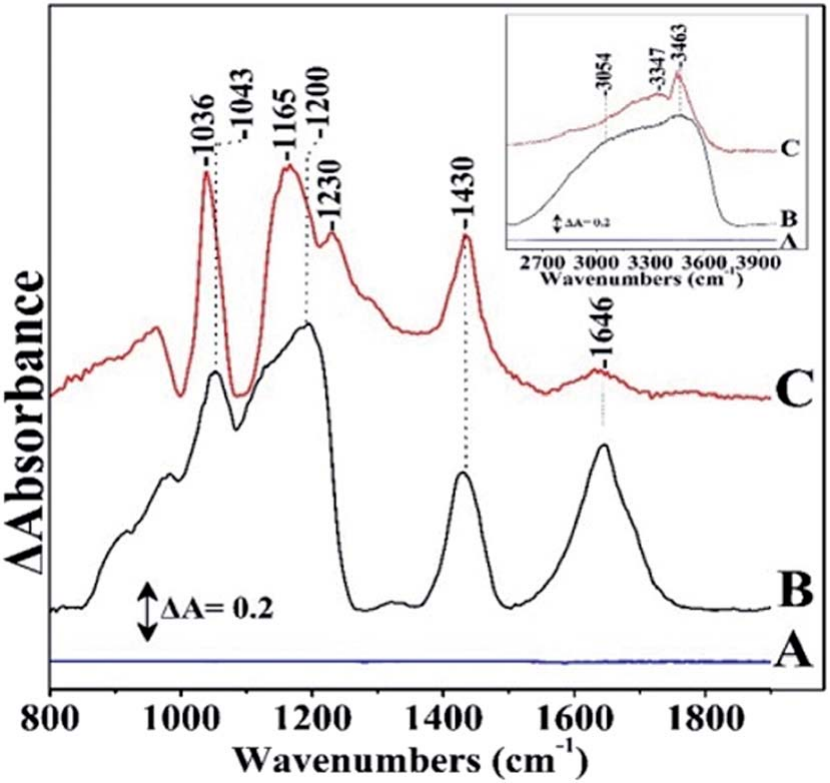
FTIR spectra of (A) graphite sheets, (B) pristine *S. thermosulfidooxidans*, and (C) *S. thermosulfidooxidans*–graphite surface.

The FTIR spectrum of graphite sheets is shown in Fig. 3A without any noticeable peaks. The FTIR spectrum of *S. thermosulfidooxidans* consists of the amide I band at 1646 cm^−1^ which is sensitive to conformational effects and the peaks ranging from 900 to 1200 cm^−1^ which have been attributed to the C–O stretch of bacterial polysaccharides, the P=O stretching of the phosphate group at 1128 cm^−1^ and 1240 cm^−1^, the symmetric stretching of O–P–C at 1057 cm^−1^ and the asymmetric ester O–P–O stretching of O–P–C modes from nuclei acids at 970 cm^−1^. The FTIR bands shown in Fig. 3 spectrum B at 1036 and 1065 cm^−1^ are due to C–O stretch of bacterial polysaccharides.^5,6^ The amide I of the pristine *S. thermosulfidooxidans* has lost most of its intensity in the *S. thermosulfidooxidans*–graphite sheets indicating that proteins are involved in the adsorption *S. thermosulfidooxidans* on the graphite surface. There are additional vibrations in the 1040–1129 cm^−1^ range due to the formation of biofilm and at 1423 cm^−1^ due to NH_3_.^5,6^ The dynamics of the biofilm is clearly demonstrated by the intensity changes/frequency shifts in the entire 900–1300 cm^−1^ region.^7,8,17^ Therefore, the developed biofilm architecture on the surfaces is heterogenous both in space and time, constantly changing because of external and internal processes.^17^ The intensity of all bands increases faster than the decay of the amide I indicating in addition to functional biofilm dynamics also an overproduction of extracellular polysaccharides with time.^17^ In addition, the band intensity increases and changes occurred in the O–P–O group spectral profile which reflects structural and compositional changes in the biofilm. In the inset spectrum are the corresponding spectra of Fig. 3 in the 2600–4000 cm^−1^ frequency range. In spectrum B the broad peaks in the 3400 cm^−1^ range are due to H_2_O and in spectrum C the bands at 3463 and 3347 cm^−1^ with correspond to O–H groups of the formed *S. thermosulfidooxidans* graphite surface. Given that graphite has no bands in that frequency range and the bands of H_2_O in spectrum B are very broad we suggest that the observed bands in spectrum C at 3424–3436 cm^−1^ arise from the stretching vibration of –OH groups (V_O–H_) either from –OH groups of absorbed water or –OH groups formed during the oxidation processes.

### 3.4 Electron transfer processes. Direct EET *via* outer membrane redox proteins

Bacterial adhesion is mediated by a multitude of specific and nonspecific forces, associated with the constituents of the bacterial surface. Besides specific forces, non-specific forces such as hydrophobic interactions and electrostatic interactions also contribute to bacterial adhesion.^6^ We propose that carboxylic residues of proteins *via* the COO(H) groups that can form H-bonds with both the C and/or the O(H) may specifically bond with H_2_O to induce initial adhesion of *S. thermosulfidooxidans* to the carbon atoms of graphite. After initial adhesion or contact the planktonic cells of *S. thermosulfidooxidans* adapt to the new environment by enhancing adhesion or contact to form biofilms. In the long term, the EPS composition is changed and more stable bonds occur. In this case, we propose that the blue copper protein sulfocyanin which is a component of the electron transport chain in iron oxidation of *S. thermosulfidooxidans*, plays a significant role as the primary electron acceptor from the surface of the graphite resulting in the formation of GO.

It is known that some microorganisms excrete organic compounds to form a conditioning film on solid surfaces.^7,8^ This film is able to modify the solid surface properties and, thus, change the subsequent adhesion and biofilm formation. In this context, a substantial amount of EPS excreted into the solution which may have been detected as colloidal EPS, this may function to form a conditioning film actively modifying the graphite surface. We propose that the primary step in the oxidation of graphite by *S. thermosulfidooxidans* is the intercalation of water on the surface of graphite followed by the formation of EPS on the surface of the graphite sheets. The transformation of epoxy groups to hydroxyl forms hydrogen bonds between these groups and other water molecules, which additionally facilitates the intercalation process. Our data are consistent with direct contact mechanism which is based on the attachment of *S. thermosulfidooxidans* to the graphite sheets surface. The bio-processes, resulting in the oxidation of graphite sheets producing GO sheets, occur at the interface between the organism and the graphite sheets surface. We propose that the attached cells are embedded in EPS on the graphite sheet surface. Iron(iii) ions from the bacteria growth medium complexed in the EPS facilitate the oxidation of the graphite sheets. Produced iron(ii) ions are directly re-oxidized by the attached bacteria through the copper protein sulfocyanin to iron(iii). Thereby, the attacking agent is regenerated. This way, GO sheets which are of great significance for the preparation of graphene materials with specific properties can be prepared.^18–20^

## 4. Conclusions

In this work, we demonstrate by Raman^21,22^ and FTIR^23,24^ spectroscopies Microbial Extracellular Electron Transfer (MEET) for the oxidation of graphite sheets by *Sulfobacillus thermosulfidooxidans*. The observed Raman *I*_D_/*I*_G_ = 0.3 ratio is the highest defect content in the carbon lattice reported by bio-oxidation process and provides insights in the extracellular transfer of electrons by selected bacteria that occurs in microbial fuel cells.

## Conflicts of interest

There are no conflicts to declare.

## Supplementary Material
